# A comprehensive CHO SWATH-MS spectral library for robust quantitative profiling of 10,000 proteins

**DOI:** 10.1038/s41597-020-00594-z

**Published:** 2020-08-11

**Authors:** Kae Hwan Sim, Lillian Chia-Yi Liu, Hwee Tong Tan, Kelly Tan, Daniel Ng, Wei Zhang, Yuansheng Yang, Stephen Tate, Xuezhi Bi

**Affiliations:** 1 Bioprocessing Technology Institute (BTI), Agency for Science, Technology and Research (A*STAR), Singapore, 138668 Singapore; 2 SCIEX, Concord, Ontario, Canada; 3 Duke-NUS Medical School, Singapore, 169857 Singapore

**Keywords:** Proteomic analysis, Expression systems, Mass spectrometry, Proteomics

## Abstract

Sequential window acquisition of all theoretical fragment-ion spectra (SWATH) is a data-independent acquisition (DIA) strategy that requires a specific spectral library to generate unbiased and consistent quantitative data matrices of all peptides. SWATH-MS is a promising approach for in-depth proteomic profiling of Chinese hamster Ovary (CHO) cell lines, improving mechanistic understanding of process optimization, and real-time monitoring of process parameters in biologics R&D and manufacturing. However, no spectral library for CHO cells is publicly available. Here we present a comprehensive CHO global spectral library to measure the abundance of more than 10,000 proteins consisting of 199,102 identified peptides from a CHO-K1 cell proteome. The robustness, accuracy and consistency of the spectral library were validated for high confidence in protein identification and reproducible quantification in different CHO-derived cell lines, instrumental setups and downstream processing samples. The availability of a comprehensive SWATH CHO global spectral library will facilitate detailed characterization of upstream and downstream processes, as well as quality by design (QbD) in biomanufacturing. The data have been deposited to ProteomeXchange (PXD016047).

## Abstract (summary)

**Table:** 

Measurement(s)	Proteomic Profile • database type spectral library
Technology Type(s)	mass spectrometry • SWATH MS protein profiling assay • computational modeling technique
Sample Characteristic - Organism	Cricetulus griseus

Machine-accessible metadata file describing the reported data: 10.6084/m9.figshare.12480386

## Background & Summary

Chinese hamster ovary (CHO) cells are widely studied in biomedical research and have been used for the production of nearly 70% recombinant therapeutic proteins, including the blockbuster monoclonal antibodies (mAbs) such as adalimumab (Humira), bevacizumab (Avastin), and trastuzumab (Herceptin)^1–4^. As with other biopharmaceuticals, the production of recombinant mAbs using CHO cells is strictly regulated by the US Food and Drug Administration (FDA) and the European Medicines Agency (EMA)^5^. The quality by design (QbD) approach is emphasized in order to ensure all aspects of CHO mAb product development and manufacturing are evaluated and consistent^6–9^. Hence, in order to enhance the mAb product quality while minimizing potential adverse effects, a thorough understanding of the recombinant mAbs, the early-stage product development process as well as the production and purification process is critical.

Mass spectrometry (MS)-based proteomics techniques have been applied to facilitate the QbD approach for optimal bioprocess design, and enhanced quality and yield of biotherapeutic products^10–13^. For example, the data-dependent acquisition (DDA) shotgun proteomics has been demonstrated to complement the traditional immunoassays (western blotting and ELISA) to monitor and analyze known and/or previously unseen host cell proteins (HCPs) in the drug manufacturing processes^14–16^. Nevertheless, due to the stochastic nature of peptide sampling by DDA-MS technique, multi-dimensional separation or fractionation steps and lengthy chromatographic gradients are often required to increase the proteome coverage over a large dynamic range of protein concentrations. Additionally, the advanced MS methodologies usually require well-trained professionals to operate the instrument and analyze the MS data in a consistent manner. These factors limit the throughput and militate the viability of MS-based techniques as a routine method for bioprocess development and optimization.

Sequential window acquisition of all theoretical fragment-ion spectra (SWATH), a specific variant of data-independent acquisition (DIA)-based MS technique introduced by SCIEX, provides the possibility to overcome the limitations imposed by traditional DDA-based MS techniques^17–19^. In SWATH-MS, all ionized compounds of a given sample within a specified mass range (window) are fragmented in a systematic and unbiased fashion through a sequential series of predefined precursor isolation windows. The complete information of precursors and product ions will be permanently recorded in a single scan, offering highly specific and multiplexed MS data to quantitate the analytes in a manner equivalent to selected reaction monitoring (SRM). Furthermore, SWATH-MS coupled with peptide-centric data extraction strategy enables quantification of multiple analytes, and this has recently been demonstrated in bioprocessing research^20–23^. Importantly, a specific spectral library containing the empirically accurate SWATH assay coordinates is preferred over the computational prediction methods (library-free analysis) to specifically extract high-quality quantitative measurements from the SWATH-MS data^23–27^. However, no CHO spectral library has been publicly available thus far; the potential commercial value and competing interest may have prevented the release of the in-house spectral libraries generated by the biopharmaceutical companies and contract research organizations. With the aim to facilitate and improve the consistency in bioprocess development as well as to benefit the academic research, we believe that the construction of a publicly accessible CHO-specific SWATH-MS spectral library is significant for future SWATH-MS applications in biopharma R&D and biomanufacturing.

Here, we present a comprehensive SWATH-MS CHO global spectral library generated through a series of systematic analyses of mAb-producing CHO-K1 intracellular proteome using advanced LC-MS technique coupled with multi-dimensional fractionation strategies. More than 10,000 proteins were identified in the library, and stringent filtering criteria were implemented in the subsequent fine-tuning steps to include only the assays which are reliably detected. The robustness, accuracy and consistency of the CHO global spectral library were demonstrated by SWATH-MS analyses of different samples obtained from CHO whole cell lysates (WCL), harvested cell culture fluids (HCCF) and downstream processing (DSP) mAb samples, and across multiple instrumental setups. These results have also shown the feasibility of SWATH-MS with the in-house CHO global spectral library as a potential process analytical technology (PAT) to resolve the bioprocessing issues within the QbD paradigm for future biopharmaceuticals manufacturing system. The original MS data and the CHO spectral library demonstrated in current study are deposited to the ProteomeXchange (PXD016047)^28^.

## Methods

### Reagents

Reagents used in the experiments were purchased from Sigma-Aldrich (St Louis, MO, US) unless stated otherwise.

### CHO cell cultures

The mAb-producing CHO-K1 (CCL-61, ATCC) cells were cultured in fully chemically-defined protein-free culture medium as described previously^29^. The cells were subcultured every 3 days in 125 mL disposable Erlenmeyer shake flasks (Corning, Acton, MA), and incubated on a shaker platform at 110 rpm in a humidified 37 °C / 8% CO_2_ incubator. Viable cell density and viability were measured by Vi-CELL^TM^ XR 2.04 cell viability analyzer (Beckman Coulter, Brea, California) according to the manufacturer’s instructions. Harvested cell culture fluid (HCCF, collected on day 14 of CHO-K1 fed-batch cell cultures), CHO-DG44 and CHO-S cell cultures were obtained from the Animal Cell Technology group in Bioprocessing Technology Institute (BTI), A*STAR, Singapore.

### Protein sample preparation and enzymatic digestion

**Fig. 1:**
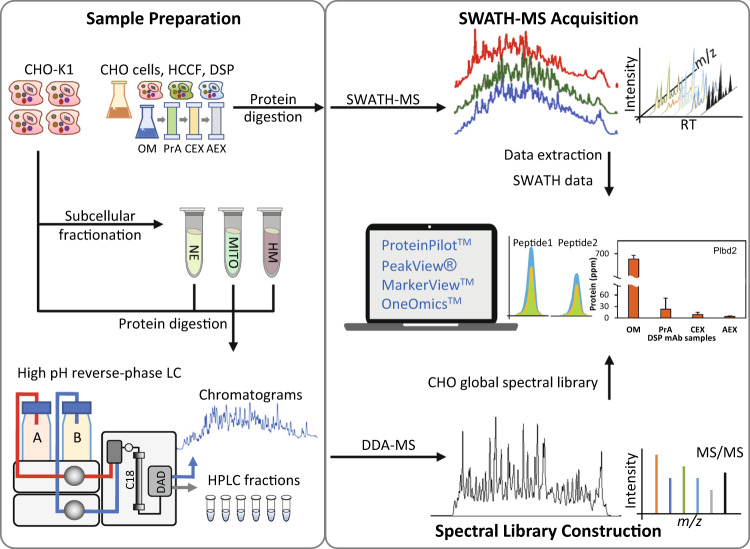
Workflow for creating and using the SWATH CHO global spectral library. The CHO-derived samples were processed using in-house multi-dimensional separation protocol. Briefly, the CHO-K1 cells were lysed and fractionated using differential ultracentrifugation to isolate nuclear (NE), mitochondrial (MITO), and heavy-membrane (HM) compartments. The protein lysates from whole cell (WCL) and subcellular-organelle compartments were tryptic digested, subsequently fractionated using basic reverse-phase liquid chromatography separation, and subjected to DDA-MS analysis. Protein digest from harvested cell culture fluid (HCCF) and downstream processing (DSP) mAb samples were directly subject to SWATH-MS in TripleTOF 6600. The raw DDA data was searched locally in ProteinPilot^TM^ software and the results were uploaded to OneOmics^TM^ for spectral library construction. The SWATH-MS data sets were processed locally using PeakView® and MarkerView^TM^ or using OneOmics^TM^. The applicability and robustness of the CHO global spectral library were evaluated with SWATH-MS data sets of different CHO-derived samples, including WCL of different cell lines, HCCF and DSP mAb samples, and using various LC-MS instrumental setups.

CHO cells were pelleted at 1,500 *g*, washed thrice with ice-cold phosphate buffered saline (PBS) and re-suspended in a SDS cell lysis buffer containing 50 mM triethylammonium bicarbonate buffer (TEAB) pH 8.5, 5% SDS, and 1x Halt^TM^ protease inhibitor cocktail (Thermo Fisher Scientific, Waltham, MA). The cell lysates were further disrupted using an UP50H ultrasonic processor (Hielscher Inc. Teltow, Germany) at 30% amplitude, with 0.5 s pulse on and 0.5 s pulse off, for 20 times on ice, and clarified by centrifugation at 20,000 *g* for 10 min. Protein concentration was determined using the BCA Protein Assay Kit (Pierce^TM^, Thermo Fisher Scientific) according to the manufacturer’s instructions. The DSP mAb samples were processed using a typical DSP purification procedures (Fig. 1): starting with clarified HCCF original material (OM), mAb was captured with protein A affinity chromatography using MabSelect SuRe LX resin (GE Healthcare, Uppsala, Sweden), followed by cation exchange chromatography for intermediate purification using POROS XS resin (Thermo Fisher Scientific, Waltham, MA) with salt gradient elution, and anion exchange chromatography for polishing using POROS HQ resin (Thermo Fisher Scientific, Waltham, MA) in a flow-through mode.

mAb concentrations were measured by analytical SEC with a TSKgel G3000SWXL column (Tosoh Bioscience, South San Francisco, CA) on a Dionex UltiMate^TM^ 3000 HPLC system (Thermo Fisher Scientific, Waltham, MA) operated at a flow rate of 0.6 mL/min, using a buffer with the formulation of 50 mM MES, 20 mM EDTA, 200 mM arginine, pH 6.5. The sample injection volume was 100 µL. mAb IgG concentrations were calculated by comparing the experimental results with a calibration curve prepared from the known concentrations of purified mAb, determined by SoloVPE (C Technologies, Inc. Bridgewater Township, NJ). HCP content in DSP samples was determined by ELISA using a Generation III CHO HCP kit (Cygnus Technologies, Southport, NC) according to the manufacturer’s instructions.

HCCF and DSP mAb samples obtained from CHO-K1 were concentrated using 10,000 MWCO Vivaspin^®^ 20 centrifugal concentrators (#VS2002), (Sartorius, Göttingen, Germany), and the proteins were precipitated using methanol-chloroform precipitation method as described previously^30^.

Enzymatic digestion: 200 µg protein of each sample was reduced and alkylated, followed by digestion on S-Trap^TM^ mini spin columns (ProtiFi^TM^, Farmingdale, NY) using Trypsin Gold, MS-grade (Promega, Madison, Wisconsin) according to the manufacturer’s protocol^31^. The eluted peptide mixtures were dried in a SpeedVac vacuum concentrator at room temperature and stored at −80 °C for future use.

### Subcellular organelle fractionation of CHO-K1

CHO-K1 cell pellets were resuspended in cell lysis buffer containing 250 mM sucrose, 20 mM HEPES-NaOH (pH 7.9), 10 mM KCl, 1.5 mM MgCl_2_, 1 mM EDTA, 1 mM EGTA, and 1x Halt^TM^ protease inhibitor cocktail. The cells were incubated on ice for 5 min with occasional vortex followed by passing through 27-gauge needle for lysate homogenization. The cell lysate was centrifuged at 800 *g* for 10 min at 4 °C to pellet down the nuclear fraction, cell debris and unbroken cells. The supernatant was collected and centrifuged at 10,000 *g* for 20 min at 4 °C to pellet the mitochondrial fraction. The supernatant was further centrifuged at 100,000 *g* for 60 min at 4 °C using a micro-ultracentrifuge (#CS150FNX) (Hitachi, Tokyo, Japan). The pellet obtained was heavy-membrane fraction (mainly rough endoplasmic reticulum) while the supernatant was cytosolic fraction. The nuclear, mitochondrial and heavy-membrane fractions were tryptic digested using the S-Trap aided protein digestion protocol.

### High pH reverse phase liquid chromatography (bRPLC) peptide fractionation

A total of 200 µg of peptide samples from CHO-K1 WCL and subcellular-organelle fractions were each subjected to a 60-min multi-step gradient performed on a Kinetex core shell C18 column (2.6 µm, EVO C18, 150 mm × 3.0 mm, 100 Å, Phenomenex, Brechbuhler AG, Schlieren, Switzerland) using an UltiMate 3000 UHPLC system (Thermo Fisher Scientific). The mobile phase was 10 mM ammonium formate (buffer A, adjusted to pH 10 using ammonium hydroxide) and 10 mM ammonium formate in 80% acetonitrile (ACN) (buffer B, adjusted to pH 10 using ammonium hydroxide). The peptide samples were separated with a gradient from 0–35% buffer B for 55 min at a flow rate of 0.8 mL/min. The fractions were collected and pooled in concatenation into 30 fractions for CHO-K1 WCL; and 10 fractions for each subcellular-organelle sample. The fractions were dried in a SpeedVac vacuum concentrator at room temperature and stored at −80 °C for future use.

### Data-dependent acquisition (DDA) – MS

For spectral library generation, estimated 1 µg of peptide mixtures from each fraction were analyzed on a TripleTOF 6600 mass spectrometer (SCIEX) coupled to a NanoSpray III Ion Source (SCIEX) and interfaced with a Waters nanoAcquity UPLC system (Waters, Milford, MA) or Eksigent Ekspert nanoLC 425 (NanoLC Ultra 2D, Eksigent, Toronto, Canada). The peptide samples were separated at 50 °C on an ACQUITY UPLC M-class peptide BEH C18 column (75 µm x 200 mm, particle size of 1.7 µm, and pore size of 130 Å). The iRT reference peptides (Biognosys AG, Schlieren, Switzerland) were added to all samples at 1:10 ratio prior to MS injection for retention time calibration. The LC system was operated with 0.1% formic acid (FA) in water (buffer A) and 0.1% FA in ACN (buffer B) at a flow rate of 300 nL/min. The separation gradient was from 5–35% of buffer B over the period of 110 min. The MS was operated in DDA top 20 mode with the following parameters: MS1 spectra were collected at 400–1,500 *m*/*z* for 500 ms, 20 most intense precursors with charge states 2–5 that exceeded 125 counts/s were selected for fragmentation, and the corresponding fragmentation MS2 spectra were collected at 50–2,000 *m/z* for 100 ms. Rolling collision energy (equation: (0).0625 × *m/z* – 10.5) (derived from SCIEX) with a collision energy spread (CES) of 15 eV was set as the fragmentation patterns used in SWATH-MS analysis.

### Data-independent acquisition (DIA) SWATH-MS

SWATH-MS acquisition was operated using the same LC-MS instrumental setup as described above with some modifications. Briefly, a 100-variable-window setup was generated using the SWATH® Variable Window Calculator 1.1 (SCIEX) with a 1 *m/z* window overlap on the lower side of the window. The MS1 survey scan was acquired from 400–1,250 *m/z* for 250 ms and MS2 spectra were acquired in high-sensitivity mode from 100–1,500 *m/z* for 30 ms. The total cycle time was ~3.3 s. The collision energy used in SWATH-MS acquisition was that applied to a doubly charged precursor centered in the middle of the isolation window calculated with the same collision energy equation for DDA, and with a CES of 5 eV. For the analyses conducted in capillary flow and microflow rate, the SWATH-MS data were recorded on a TripleTOF 6600 mass spectrometer coupled to a DuoSpray Analytical Ion Source (SCIEX). For capillary flow LC setup, the Eksigent Ekspert nanoLC 425 system was connected to a Waters CSH C18 column (300 µm × 150 mm, 1.7 µm, 130 Å) or a Eksigent ChromXP C18 column (300 µm × 150 mm, 3 µm, 120 Å) and operated in trap-elute mode for 1-h gradient at a flow rate of 5 µL/min. For the microflow LC setup, a Waters nanoAcquity LC system connected with a Waters CSH C18 column (1 mm × 150 mm, 1.7 µm, 130 Å) was utilized and the system was operated in direct-inject mode at a flow rate of 50 µL/min. The 100-variable-windows setup was applied and optimized in both the capillary flow and microflow rate instrumental system.

### Generation of CHO spectral library

The CHO spectral library was constructed using the workflow established by SCIEX^32^. Briefly, the raw DDA data files were processed using the ProteinPilot^TM^ software (version 5.0.1) against a Chinese hamster (CH) protein sequence database. The database was the latest release of CH RefSeq assembly (downloaded on August 2019 from ncbi.nlm.nih.gov; GCF_003668045.1_CriGri-PICR_protein.faa), appended with the Biognosys iRT fusion protein sequence and in-house mAb heavy- and light-chain protein sequences. The alkylation reagent was iodoacetamide. The search effort setting was Rapid ID with carbamidomethyl (C) as a fixed modification, and oxidation (M), deamidation (NQ) and pyroglutamic acid conversion (EQ) as variable modifications. Each of the DDA raw data files was searched in ProteinPilot^TM^ and the result files were used as the input libraries for spectral library generation. The input libraries and SWATH-MS acquisition data were uploaded to the Illumina BaseSpace Cloud (www.basespace.illumina.com) using the CloudConnect software and processed in SCIEX Cloud OneOmics^TM^ (Fig. 1). For the construction of spectral library, each input library was filtered by 1% FDR and 99% confidence threshold to remove low-confidence peptide identifications. The largest filtered input library, which contained the highest number of high-confidence peptides, were selected as base, and peptides identified from smaller filtered input libraries were merged to the base library using a non-linear calibration strategy in the OneOmics^TM^^32^. Any newly identified peptides are added to the existing proteins and any newly detected proteins are added if not present in the base library. The merged spectral library was further processed with an in-house script to identify and remove any shared and/or modified peptides.

The combined spectral library was constructed by searching all the 63 DDA raw data files together in the ProteinPilot^TM^ software. The 1D spectral library was generated using unfractionated peptide mixtures from a CHO-K1 WCL sample.

### SWATH-MS data analysis

The SWATH-MS data analysis was performed in the OneOmics^TM^ or the SWATH^TM^ Processing software in local PC. In the OneOmics^TM^, the CHO endogenous peptides were extracted according to the precursor *m/z*, intensity and confidence of identification across the entire time range, and the best scoring peak groups were used for RT calibration. The data was filtered by 1% FDR and the comparisons of proteins and/or peptides were further filtered by 20% coefficient of variation (CV) cutoff between replicates. In local SWATH-MS processing workflow, the spectral library and SWATH-MS acquisition data were loaded into SWATH^TM^ Processing microApp in PeakView® software. The iRT, mAb and shortlisted endogenous CHO peptides were manually selected as reference peptides for RT calibration. The peak groups were extracted with a 99% peptide confidence threshold and a 1% peptide FDR cutoff. The RT extraction window and the fragment ion mass tolerance were set to 5 min and 75 ppm, respectively. After data extraction, the results were imported into MarkerView^TM^ (version 1.3.1) for further data processing and analysis. Microsoft^®^ Excel, Python programming and Kyoto Encyclopedia of Genes and Genomes (KEGG) analysis^33^ were also applied in the downstream data browsing and analysis.

## Data Records

The raw DDA data files for library generation, the search result (group files), the CHO spectral library, and the SWATH-MS acquisition data applied in the current study have been deposited to the ProteomeXchange Consortium (http://proteomecentral.proteomexchange.org)^34^*via* the PRIDE partner repository^35,36^ with the data set identifier PXD016047^28^.

## Technical Validation

### Error rate control during spectral library generation

The number of proteins and peptides identified from the raw DDA data files are significantly affected by the quality and redundancy of the protein sequence database used. UniProtKB/Swiss-Prot^37^, a freely accessible database of protein sequence and functional information, is manually annotated and reviewed to contain high-quality and non-redundant protein information and hence highly recommended for the purpose of public spectral library generation. However, the UniProtKB/Swiss-Prot contains only 14 and 237 reviewed entries for Chinese hamster/CHO-K1 and *Cricetulus griseus*, respectively. We therefore used the most updated NCBI reference sequence (RefSeq) proteome database of Chinese hamster in this study. NCBI RefSeq^38^ is also a curated non-redundant collection of sequences which records genomes, transcripts and proteins information from multiple sources. We searched the mass spectra from the DDA raw data against the Chinese hamster proteome database containing 46,750 protein entries. The error rate in the analysis of these large sample cohorts was carefully handled to prevent the accumulation of false positive identifications during the construction of the spectral library. We utilized the library merging algorithm established in the OneOmics^TM^ to filter the lists of peptides in ProteinPilot^TM^ group files to a 1% global protein FDR^32^. All the peptides that passed the 99% peptide confidence threshold were selected and merged to the base library using a non-linear retention time calibration strategy^32^. Any shared or modified peptides (except those with carbamidomethylation on cysteine residue) were identified and removed from the spectral library using an in-house Python script to avoid any possible redundancy and inaccuracy in SWATH-MS data extraction and quantification using the spectral library. A total of 10,974 proteins, comprised of 199,102 peptides, 247,135 precursors and 741,405 transitions, were represented in the finalized library, which was known as CHO global spectral library hereafter.

### Coverage of the CHO global spectral library

**Fig. 2:**
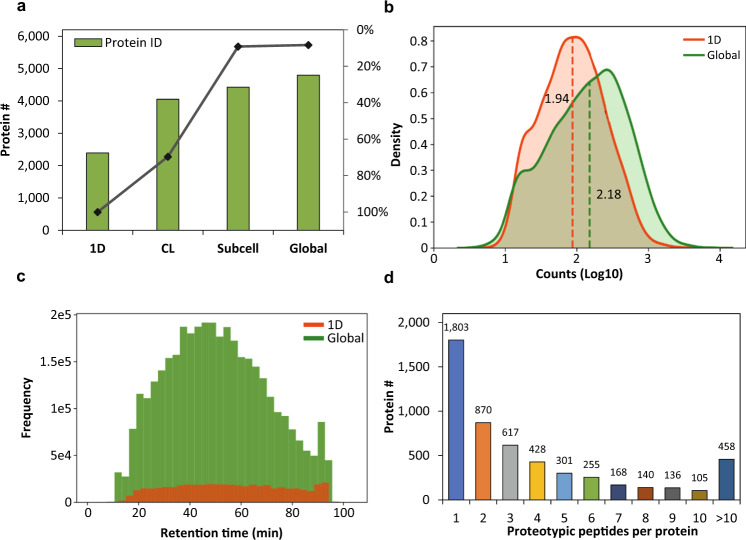
Characteristics of the SWATH CHO global spectral library. (**a**) The stacked bar chart showed the numbers of confidently quantified protein ID (at 20% CV cutoff and 1% peptide FDR) when using 1D library (1D, 3 fractions), cell-lysate library (CL, 33 fractions), subcellular-organelle library (Subcell, 33 fractions), and CHO global spectral library (Global, 63 fractions). The percentage of increase was calculated by dividing the number of increased protein ID by the total number of protein ID. (**b**) The distributions of the assays per protein in the CHO global spectral library and 1D library. The dash lines indicated the median values of the assays per protein. (**c**) The histogram distributions of the coverage and the number of peptides eluting across the LC separation gradient for the CHO global spectral library and 1D library. (**d**) The bar chart showed the confidently quantified proteotypic peptides per protein (at 20% CV cutoff and 1% peptide FDR) in the CHO-K1 WCL SWATH-MS data set using the CHO global spectral library.

During the construction of CHO global spectral library, it was found that the number of confidently quantified proteins (at 20% CV cutoff and 1% peptide FDR) reached plateau despite introducing more DDA files into the spectral library (Fig. 2a). As shown in Fig. 2a, the Subcell library (contained 30 fractions of bRPLC-separated subcellular compartments and 1D library) quantitated 9.18% more proteins than the CL library (contained 30 fractions of bRPLC-separated cell lysate and 1D library). By using the CHO global spectral library (consisted of the fractions from Subcell library, CL library and 1D library), we quantitated slightly more proteins (8.34%) than the previous work (Fig. 2a). It is noteworthy that a large number of library inputs will critically increase the time required for loading libraries into OneOmics^TM^. Therefore, we did not further fractionate the CHO-K1 protein lysate as these DDA inputs have provided sufficient CHO proteome coverage while allowing reasonable downstream SWATH-MS data processing time. In summary, the CHO global spectral library was constructed by using the MS/MS spectral data obtained from the unfractionated (1D) CHO-K1 cell lysates as the base, followed by adding the MS/MS spectral data sets derived from the three subcellular organelles (10 fractions of nuclear compartment, 10 fractions of mitochondrial compartment and 10 fractions of heavy-membrane compartments) and an extensively separated CHO-K1 whole-cell protein lysate (30 fractions).

The CHO global spectral library has reached a 23.48% proteome coverage of the 46,750 protein entries represented in the CHO RefSeq proteome database. This is 2.5 times more proteins and 8.8 times more peptides than a non-fractionated 1D spectral library (comprising of 3,134 proteins and 20,249 peptides identification) (Table 1). We also compared the CHO global spectral library (which was built based on the CHO-K1 cell proteome) to the latest proteomics study of CHO-DG44 and CHO-S cell lines (Table 1)^13^. When filtered with at least two unique peptides, the CHO global spectral library identified significantly more proteins (+56.82% to +72.42%) than individual CHO-DG44 and CHO-S data set, respectively, and a slightly higher number of proteins (+2.03%) than the combination of these two data sets. Remarkably, the CHO global spectral library represented a 242.52% to 313.03% improvement over the previous work in peptide identification (Table 1). The CHO global spectral library is thus able to provide a significantly higher number of empirically detected peptide identities for better protein identification and quantification. As displayed in Fig. 2b, the CHO global spectral library showed high similarity in the distribution of the number of assays per protein as that of 1D library, but the former covered a wider effective separation gradient with its large number of peptide identities (Fig. 2c).

**Table 1:** Comparison of the proteome coverage between CHO global spectral library, 1D library and the latest proteomic studies on CHO-DG44 and CHO-S cells.

	DDA files #	Protein #^a^	Protein #^a^ (≥2 peptides)	Peptide #^a,b^	Confidence cutoff	Data processing pipeline
CHO global spectral library	63	10,974	9,549	199,102^b^	99% peptides confidence & 1% protein FDR	ProteinPilot & OneOmics
CHO-K1 1D library	3	3,134	2,810	20,249^b^	99% peptides confidence & 1% protein FDR	ProteinPilot & OneOmics
CHO-DG44 exponential^13^	48	N.A	5,950	58,128	1% FDR	Proteome Discoverer
CHO-DG44 stationary^13^	48	N.A	5,593	50,194	1% FDR	Proteome Discoverer
CHO-S exponential^13^	48	N.A	6,089	53,958	1% FDR	Proteome Discoverer
CHO-S stationary^13^	48	N.A	5,538	48,205	1% FDR	Proteome Discoverer
Combined CHO-DG44 and CHO-S^13^	192	N.A	9,359	N.A	1% FDR	Proteome Discoverer

^a^The iRT, reverse sequences, and the proteins containing only non-CAM modified peptides were excluded.

^b^The modified peptides except for carbamidomethylation on cysteine residue were excluded.

**Fig. 3:**
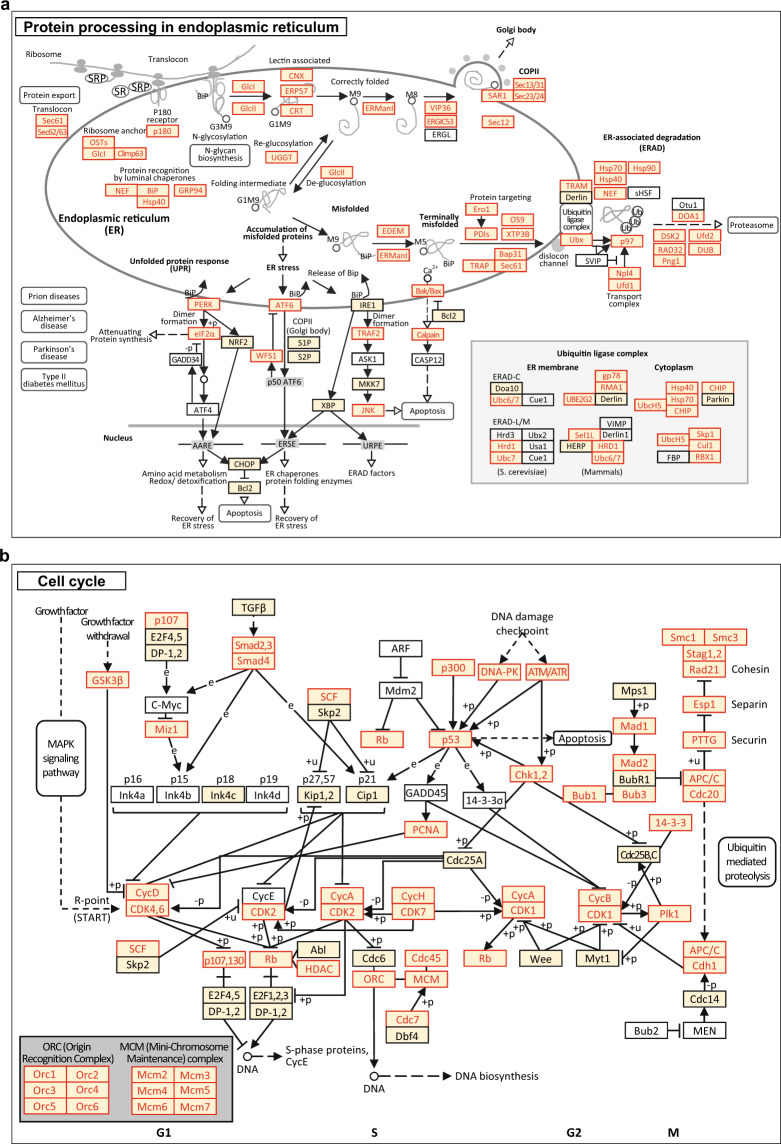
KEGG pathway mapping of CHO global spectral library. KEGG pathway analysis was applied to study the protein coverage in the (**a**) Protein processing in endoplasmic reticulum and (**b**) Cell cycle pathways. The proteins represented in the CHO global spectral library were boxed in yellow color while those confidently identified in the CHO-K1 SWATH-MS data set were highlighted in red color.

A CHO-K1 WCL SWATH-MS data set was processed with the CHO global spectra library. Up to 3,478 proteins (65.86%) could be confidently quantified (at 20% CV cutoff and 1% peptide FDR) with at least two unique peptides (Fig. 2d). Additionally, we perceived the paramount significance to quantitatively identify the critical components in CHO biological pathways relating to the stability, quality and productivity of recombinant protein production as well as the cell viability. By using KEGG pathway mapping analysis, we demonstrated that proteins associated with Protein Processing in ER (83.81%, Fig. 3a), Cell Cycle (82.98%, Fig. 3b) and N-glycan Biosynthesis (82.05%, data not shown) were well represented in the CHO global spectral library (proteins boxed in yellow color in Fig. 3). In addition, more than half of these proteins could be confidently identified (at 20% CV cutoff and 1% peptide FDR) in the SWATH-MS analysis (proteins highlighted in red color in Fig. 3).

### Calibrated retention time in CHO global spectral library

One of the advantages of adopting nanoflow instrumental setup in the construction of SWATH spectral library was that nanoflow LC-MS provides the highest sensitivity in DDA-MS without requiring huge amounts of starting materials as compared to higher flow LC-MS. However, retention time drift^39^ is an inherent issue in the nanoflow instrumental setup which would lead to retention time misalignment in combined database search (the first step in constructing spectral library). Therefore, we utilized the library merging algorithm in OneOmics^TM^ to improve the retention time correlation and alignment between input libraries during the library construction. In subsequent SWATH-MS data extraction procedure in OneOmics^TM^, a non-linear autoRT calibration algorithm was applied^32^. Briefly, one hundred high-abundance, confidently detected peptides, including those of iRT peptides, mAb protein and endogenous CHO proteins, were selected as reference peptides across the entire scan time range. The retention time frame of spectral library was matched to that of SWATH-MS data based on the reference peptides to fine-tune the retention time from run to run. Here we compared the CHO global spectral library and the uncalibrated combined-searched spectral library in the SWATH-MS analysis of CHO-K1 WCL data set. The CHO global spectral library identified a total of 6,445 proteins, which was 49.54% more than that of the combined-searched library (1% peptide FDR) (Table 2). At 20% CV cutoff, the CHO global spectral library quantitated higher numbers of protein (4,717) and peptide (16,919) identities as compared to those of the combined-searched spectral library (2,537 proteins and 8,252 peptides) (Table 2). On top of that, the former has reported lower median CV values in both protein- and peptide-level quantitation (Table 2). These results highlighted the effectiveness of retention time calibration during the library merging and data extraction procedure. By using the CHO global spectral library which contained highly accurate predicted retention times, we could execute correct and consistent SWATH-MS data analysis.

**Table 2:** Comparison of CHO global spectral library and uncalibrated combined-search spectral library in CHO-K1 WCL SWATH-MS data.

Spectral library	CHO global spectral library	Uncalibrated combined-search spectral library
Total identified proteins	6,445	4,310
20% CV cutoff	4,717	2,537
10% CV cutoff	3,183	1,414
Median CV	10.16%	15.47%
Total identified peptides	23,971	14,061
20% CV cutoff	16,919	8,252
10% CV cutoff	10,325	4,585
Median CV	11.73%	15.42%

### Portability of the CHO global spectral library

**Fig. 4:**
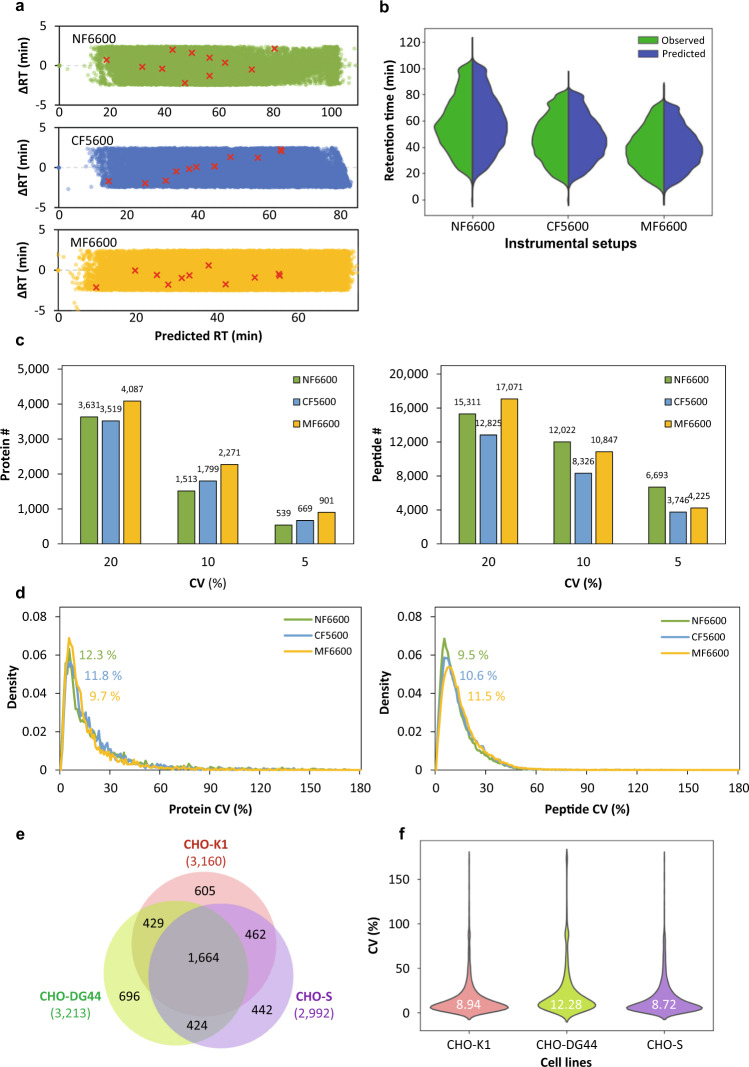
The robustness of the SWATH CHO global spectral library in quantification of CHO proteome. (**a**) The correlation between the ∆RT and the predicted retention times in three LC-MS instrumental setups. The red colored “×” represented the 11 iRT reference peptides. (**b**) The violin plot showed the distribution of observed and predicted retention times in three instrumental setups. (**c**) The bar charts showed the numbers of confidently quantified proteins (left) and peptides (right) after 20%, 10% and 5% CV cutoff. (**d**) The CV distribution of all the identified proteins (left) and peptides (right) in three LC-MS instrumental setups. The median CV values in each condition were highlighted. (**e**) Venn diagram analysis of confidently quantified protein ID (at 20% CV cutoff and 1% peptide FDR) between three CHO cell lines. The total protein IDs of respective cell lines were indicated in brackets. (**f**) The violin plot showed the distribution of the protein CV values across three CHO cell lines with the median CV values highlighted in white color. NF6600: nanoflow LC coupled to TripleTOF 6600; CF5600: capillary flow LC coupled to TripleTOF 5600 + ; MF6600: microflow LC coupled to TripleTOF 6600.

Different laboratories may acquire their SWATH-MS data sets using various instrumental setups. For example, in biopharmaceutical industries, higher flow-rate LC-MS analytical instruments are often preferred in order to achieve high reproducibility and throughput with a shorter duration of data acquisition and processing. Since the CHO global spectral library was established in nanoflow LC-MS setup (TripleTOF 6600 coupled to Waters nanoAcquity LC), we validated its performance with three CHO-K1 WCL data sets acquired in nanoflow LC (Eksigent) coupled to TripleTOF 6600 (NF6600 data), capillary flow LC (Eksigent) coupled to TripleTOF 5600+ (CF5600 data), and microflow LC (Waters) coupled to TripleTOF 6600 (MF6600 data) (Table 3). Noted that in CF5600 and MF6600 we analyzed higher loading amounts of sample to compensate for the lower sensitivity at higher flow rate LC-MS. The relationship between the delta retention times (∆RT = observed retention times – predicted retention times) and the predicted retention times were studied. As shown in Fig. 4a, the ∆RT maintained within ±2.5 minutes across the entire predicted retention time range (red colored “x” indicated the eleven iRT reference peptides). Graphical demonstration using violin plot showed the distribution of observed and predicted retention times were highly consistent in the three LC-MS setups (Fig. 4b). Total 4,222 proteins were confidently quantified from three data sets (at 20% CV cutoff and 1% peptide FDR): In NF6600 there were 3,631 proteins (15,311 peptides) quantified when 1 µg of WCL was analyzed; In CF5600 3,519 proteins (12,825 peptides) were quantifiable from 5 µg of WCL; In MF6600 up to 4,087 proteins (17,071 peptides) were quantified from 20 µg of WCL (Fig. 4c). Hundreds to thousands of proteins and peptides could be consistently quantified in three data sets when more stringent CV cutoffs (5 and 10%) were applied (Fig. 4c). Right-skewed distribution of CV values with low median CVs ranging from 9.5% to 12.3% in both the protein- and peptide-level quantification demonstrated the portability as well as the robustness of the CHO global spectral library for the analyses of SWATH-MS data sets acquired across three LC-MS setups (Fig. 4d). These results have further elaborated the effectiveness of retention time calibration in our pipeline, and supported the potential transfer of the CHO global spectral library to industrial scale application which often requires high throughput with short sample and data handling duration.

**Table 3:** Details of three LC-MS instrumental setups for the SWATH-MS acquisition of CHO-K1 WCL samples.

LC-MS Setup	NF6600 (TripleTOF 6600 coupled to nanoflow LC)	CF5600 (TripleTOF 5600 + coupled to capillary flow LC)	MF6600 (TripleTOF 6600 coupled to microflow LC)
MS	SCIEX TripleTOF 6600 (A)	SCIEX TripleTOF 5600+	SCIEX TripleTOF 6600 (B)
Acquisition mode	SWATH-MS	SWATH-MS	SWATH-MS
LC model	Eksigent Ekspert nanoLC 425 (nanoflow module)	Eksigent Ekspert nanoLC 425 (microflow module)	Water nanoAcquity UPLC (microflow)
Injection mode	Trap-elute	Trap-elute	Direct-inject
Column	Waters BEH C18 75 µm × 200 mm	Eksigent ChromXP C18 300 µm × 150 mm	Waters CSH C18 1 mm × 150 mm
Flow rate	300 nL/min	5 µL/min	50 µL/min
LC gradient	100 min	95 min	90 min
Sample amount	1 µg	5 µg	20 µg

In addition to CHO-K1 cells, there are other CHO-derived cell lines that are commonly studied and widely utilized in the biomanufacturing process. We evaluated the performance of the CHO global spectral library, which was built using the CHO-K1 cell proteome searched against the Chinese hamster RefSeq database, in the SWATH-MS analysis of another two CHO cell lines, namely CHO-DG44 and CHO-S. In this analysis, more than 4,700 proteins were confidently quantified (at 20% CV cutoff and 1% peptide FDR). A total of 1,664 proteins (35.24%) were commonly identified in the three cell lines and 2,979 proteins (63.09%) were quantified in at least two cell lines (Fig. 4e). The median CV values for the protein abundances in each cell line ranged from 8.72% to 12.28% while the median CV values for all the quantified proteins across the three cell lines was 9.97% (Fig. 4f). The SWATH-MS results of CHO-DG44 and CHO-S cells suggested that the CHO global spectral library can be effectively used to quantify thousands of proteins across multiple CHO cell lines.

### Robustness of protein identification and quantification in CHO HCCF and HCPs in DSP mAb samples

Being the most widely utilized host cell in biopharma mAb production, the systematic and comprehensive analysis of the intracellular CHO proteome using DIA SWATH-MS will help to better understand this model cell line and contribute towards the rational improvement of CHO cells performance^40^. In addition, the profiling of HCCF protein dynamics will also facilitate the bioprocess design and optimization, and the monitoring of HCP impurity in the biologics product^41^.

**Fig. 5:**
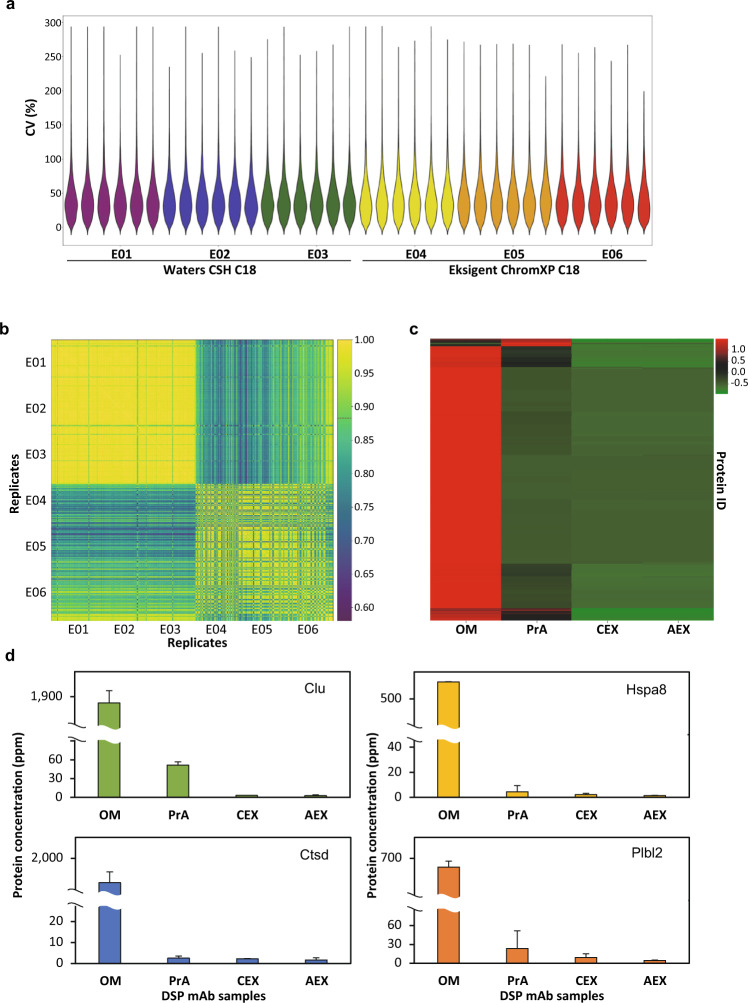
The application of CHO global spectral library in proteome profiling and quantification of HCCF and DSP mAb samples. (**a**) The violin plot showed the distribution of CV values across the large-scale analysis of HCCF samples. The CV values were calculated using the technical replicates for each of the six biological replicates in E01 – E06. (**b**) The Pearson correlation matrix of the abundance of proteins identified in the HCCF samples from experimental group E01 – E06. The median correlation coefficient r value (0.88) was represented as a red colored dash line in the color bar. (**c**) Heat map analysis of confidently identified proteins (at 20% CV cutoff and 1% peptide FDR in OM) in the typical DSP purification workflow. (**d**) The bar chart showed the average protein abundances of four well-known difficult-to-remove HCPs, including clusterin (Clu), cathepsin D (Ctsd), heat shock cognate 71 kDa protein (Hspa8) and phospholipase B-like 2 (Plbl2), identified in the DSP purification process. The error bars indicated the standard deviations between replicates. OM: original material; PrA: protein A eluate; CEX: cation exchange; AEX: anion exchange.

Subsequently, we validated the robustness of the CHO global spectral library by performing a large-scale SWATH-MS stress test experiment on unclarified HCCF samples from CHO-K1 mAb producing cultures over a period of four months. A total of 360 SWATH-MS runs were carried out in six batches of experimental groups, E01 – E03 were separated using Waters CSH C18 column; while E04 – E06 were separated using Eksigent ChromXP C18 column. Each experimental group consisted of six biological replicates (B01 – B06), and each biological replicate comprised of ten technical replicates (T01 – T10). Except for nine technical replicate runs (four from E05-B06 and five from E06-B06) which were excluded due to spectrum quality failure, total of 351 SWATH-MS data sets were analyzed in OneOmics^TM^ using the abovementioned workflow, and total 4,985 proteins were identified (at 1% peptide FDR) after SWATH-MS data extraction. The CV values for each of the six biological replicates in E01 – E06 were also determined. The violin plot showed highly similar pattern of CV distributions (median CV values of ~35%) although the MS data was acquired over a long period of four months, and two analytical columns from different vendors were used (Fig. 5a). Higher median CVs observed in this experiment might be due to the inherently higher margin of error for quantifying low abundance proteins^42^ and much wider dynamic range of protein abundances in the HCCF samples as compared to that of cell lysate samples. We have also observed that lower median CV values (~22%) is achievable when subsets of the replicates were acquired within a shorter duration (data not shown). A Pearson correlation matrix was constructed using the intensities of all identified proteins. While E01 – E03 (180 runs) and E04 – E06 (171 runs) were visually distinct from each other, corresponding to the use of two different analytical columns, the Pearson correlation matrix showed that the 351 runs were positively correlated with each other with a median correlation coefficient r value of 0.88 (indicated as a red colored dash line on the color bar in Fig. 5b and Supplementary Table 1). These results have demonstrated that extracellular CHO proteins can be identified and quantified when performing the SWATH-MS analyses of HCCF using the CHO global spectral library in OneOmics^TM^.

We next evaluated the performance of CHO global spectral library in monitoring HCP clearance in the DSP workflow. The DSP mAb samples collected from a generic DSP practice, including original material (OM), post protein A eluate (PrA), post cation exchange flow through (CEX) and post anion exchange flow through (AEX), were digested and analyzed accordingly (Fig. 1). ELISA analysis of these samples showed that the total HCP concentrations were greatly reduced from 510,762 ppm in OM, to 1,081 ppm in PrA, 557 ppm in CEX, and finally only 7 ppm in the AEX. In the SWATH-MS analysis, total 1,900 proteins were quantified (at 20% CV cutoff and 1% peptide FDR in OM) from one microgram of each DSP sample, including previously reported difficult-to-remove and problematic HCPs^43–45^ (Online-only Table 1). Heat map analysis of all the identified proteins showed that majority of the impurities were removed after protein A affinity chromatography (Fig. 5c). Although some HCPs were found to be enriched together with mAb (at the top and bottom of heat map of PrA), these impurities were properly removed by the following polishing steps to generate a highly purified sample (AEX) (Fig. 5c). The protein concentration (in ppm) of four well-known difficult-to-remove HCPs, including clusterin (Clu), cathepsin D (Ctsd), heat shock cognate 71 kDa protein (Hspa8) and phospholipase B-like 2 (Plbl2), were illustrated as examples: these HCPs were successfully removed after the DSP purification steps (Fig. 5d). The SWATH-MS results were not only in line with the ELISA result, but also provided critical information to monitor individual HCP abundance in each step of DSP purification. Taken all together, the CHO global spectral library can be effectively applied in the identification and quantification of thousands of proteins from CHO-derived samples in our SWATH-MS analysis pipeline.

## Usage Notes

### Generating alternative SWATH-MS spectral library

The current CHO spectral library has been constructed using SCIEX spectral library generation pipeline and could be directly applied in SCIEX SWATH^TM^ Processing software and OneOmics^TM^. With the library merging strategy implemented in the OneOmics^TM^, users could easily expand the proteome coverage of spectral libraries in future. However, future efforts are necessary to expand the library to include cell line-specific protein or peptide identification using other CHO cell lines.

### Application of the CHO spectral library in other LC-MS instruments

The CHO spectral library has been successfully applied in the analyses of SWATH-MS data sets acquired using different LC-MS instruments (TripleTOF 5600+) from the same vendor. To apply the spectral library in the DIA analysis using a LC-MS instrument provided by a different vendor, a user needs to ensure the similarity of the ion fragmentation and the optimization of collision energy settings when setting up a DIA acquisition method. Besides, it is recommended to use an analytical column of similar property and spike iRT peptides into the samples for accurate RT alignment and calibration in subsequent data processing.

### Absolute protein quantification of HCPs

It is feasible to perform absolute HCP quantification using the CHO global spectral library in the SWATH-MS analysis pipeline. Internal standard proteins with known concentration relative to the mAb product are spiked into the samples prior to LC-MS injection. During data analysis, the CHO global spectral library, appended with the assays of standard proteins, will be used in OneOmics^TM^. The absolute protein concentration (ppm) of targeted HCPs can then be obtained by directly comparing the protein abundance of HCPs to a calibration curve constructed from the standard proteins.

### Supplementary information

Supplementary Table 1

## Online-only Tables

**Online-only Table 1:** The protein areas of the 152 HCPs quantified in the DSP mAb samples.

Protein Id	Protein Description	MW[Cal.]	pI	OM_MEAN	OM_STDEV	PrA_MEAN	PrA_STDEV	CEX_MEAN	CEX_STDEV	AEX_MEAN	AEX_STDEV
NP_001230908.1	Tubulin alpha-1b chain	49872.57	4.94	3.32E + 05	3.27E + 03	7.22E + 03	6.02E + 03	4.89E + 03	2.63E + 03	5.02E + 03	7.77E + 02
NP_001230912.1	Aminoacyl trna synthase complex-interacting multifunctional protein 2	35386.3	7.69	4.49E + 04	2.56E + 03	9.17E + 02	2.03E + 02	1.21E + 02	1.51E + 02	8.43E + 01	1.03E + 02
NP_001230968.1	Elongation factor 2	94879.53	6.41	2.76E + 05	1.25E + 03	7.25E + 03	3.12E + 03	6.66E + 03	2.89E + 02	3.30E + 04	3.47E + 04
NP_001231030.1	Tubulin beta-5 chain	49098.5	4.78	1.74E + 05	6.85E + 03	1.53E + 04	4.50E + 03	8.98E + 03	1.86E + 03	3.73E + 03	5.01E + 02
NP_001231196.1	Aminoacyl trna synthase complex-interacting multifunctional protein 1	39699.01	9.37	7.32E + 04	2.43E + 03	4.62E + 03	1.54E + 03	4.15E + 03	3.58E + 02	1.78E + 04	3.30E + 03
NP_001231202.1	Golgi apparatus protein 1 precursor	128275.35	6.34	1.09E + 05	1.25E + 03	4.90E + 03	2.88E + 03	9.20E + 03	3.19E + 03	1.82E + 03	7.37E + 02
NP_001231331.1	Elongation factor 1-alpha 1	51088.98	9.1	9.01E + 05	2.23E + 04	4.14E + 04	4.97E + 03	3.96E + 03	1.43E + 03	4.94E + 03	1.11E + 03
NP_001231783.1	Glyceraldehyde-3-phosphate dehydrogenase	36823.87	8.49	3.71E + 06	5.56E + 04	3.12E + 03	9.96E + 02	3.64E + 03	1.26E + 03	4.50E + 03	1.84E + 03
NP_001233603.1	Beta-2-microglobulin precursor	13159.28	6.89	3.73E + 05	7.79E + 03	2.49E + 03	3.94E + 02	2.36E + 03	7.15E + 02	2.67E + 03	4.81E + 02
NP_001233622.2	Protein disulfide-isomerase precursor	56286.34	4.77	1.52E + 06	0.00E + 00	4.46E + 03	1.69E + 03	3.17E + 03	1.35E + 03	2.55E + 03	4.42E + 02
NP_001233630.1	Guanine nucleotide-binding protein g(i)/g(s)/g(t) subunit beta-1	37597.95	5.61	2.01E + 04	2.05E + 02	1.16E + 03	1.36E + 02	2.89E + 03	2.74E + 03	9.19E + 02	3.78E + 02
NP_001233634.1	Trifunctional purine biosynthetic protein adenosine-3	111688.02	6.45	1.21E + 05	1.63E + 03	3.00E + 03	9.37E + 02	6.61E + 03	2.50E + 03	4.98E + 03	3.23E + 03
NP_001233654.1	Phosphoglycerate kinase 1	46112.78	8.02	4.09E + 05	9.42E + 03	7.58E + 03	3.47E + 03	5.90E + 03	3.62E + 03	1.21E + 04	8.00E + 03
NP_001233658.1	Heat shock cognate 71 kda protein	71436.1	5.24	9.90E + 05	1.70E + 03	7.60E + 03	8.28E + 03	3.71E + 03	1.67E + 03	2.60E + 03	3.55E + 02
NP_001233662.1	T-complex protein 1 subunit alpha	61483.7	5.71	4.73E + 05	4.55E + 03	1.17E + 04	2.24E + 03	2.75E + 03	1.15E + 03	4.32E + 03	2.34E + 03
NP_001233668.1	Endoplasmic reticulum chaperone bip precursor	72320.76	5.07	6.88E + 06	1.36E + 05	8.76E + 03	5.77E + 03	2.19E + 04	2.21E + 04	8.08E + 03	3.65E + 03
NP_001233670.1	Hypoxia up-regulated protein 1 precursor	110471.62	5.09	3.43E + 05	7.59E + 03	5.95E + 03	1.69E + 03	6.10E + 03	1.63E + 03	1.03E + 04	2.59E + 03
NP_001233673.1	Glutathione s-transferase p 1	23222.26	7.64	1.62E + 06	4.19E + 04	9.83E + 03	2.55E + 03	2.60E + 03	8.25E + 02	6.29E + 03	9.27E + 02
NP_001233694.1	Peroxiredoxin-1	22005.86	8.22	8.50E + 06	8.64E + 04	3.60E + 04	1.86E + 04	9.51E + 03	2.96E + 03	6.97E + 03	4.04E + 03
NP_001233703.1	Protein disulfide-isomerase a3 precursor	55844.01	5.98	7.40E + 05	1.00E + 04	1.79E + 03	7.72E + 01	3.03E + 03	2.08E + 03	2.21E + 03	5.15E + 02
NP_001233750.1	Heat shock protein hsp 90-alpha	81056.75	4.96	8.92E + 05	2.16E + 03	6.99E + 03	2.39E + 03	8.07E + 03	2.29E + 03	3.73E + 03	1.68E + 03
XP_027243210.1	Heat shock protein hsp 90-beta isoform x11	80061.51	4.97	2.74E + 06	1.63E + 04	2.50E + 04	1.98E + 04	1.10E + 04	7.37E + 03	7.83E + 03	7.34E + 03
XP_027243827.1	T-complex protein 1 subunit delta	59603.81	8.24	4.07E + 05	3.40E + 03	8.46E + 03	2.71E + 03	8.78E + 03	3.65E + 03	2.24E + 03	7.59E + 02
XP_027243857.1	Malate dehydrogenase, cytoplasmic	36934.45	6.17	3.58E + 05	9.43E + 03	3.64E + 03	1.53E + 03	2.93E + 03	4.38E + 02	2.79E + 03	9.83E + 02
XP_027243958.1	Peptidyl-prolyl cis-trans isomerase a isoform x2	18135.48	8.44	5.95E + 05	9.10E + 03	1.92E + 03	7.67E + 02	3.08E + 03	1.31E + 03	6.03E + 03	3.23E + 03
XP_027244682.1	Lipoprotein lipase isoform x2	52415.96	7.94	1.39E + 05	2.87E + 03	1.30E + 04	7.08E + 03	7.10E + 03	8.53E + 02	6.92E + 03	3.47E + 03
XP_027245386.1	Transketolase	68892.71	7.56	7.23E + 05	6.94E + 03	8.12E + 03	4.97E + 03	5.61E + 03	1.13E + 03	5.71E + 03	2.30E + 03
XP_027246103.1	Clusterin	49430.24	5.52	3.08E + 06	1.64E + 05	8.56E + 04	8.95E + 03	5.42E + 03	3.52E + 02	4.63E + 03	2.33E + 03
XP_027246161.1	Cathepsin b	37487.37	5.73	1.61E + 06	2.16E + 04	7.52E + 03	7.48E + 03	9.78E + 03	2.58E + 03	3.36E + 03	1.06E + 03
XP_027246369.1	Proteasome activator complex subunit 2	26429.15	5.56	1.13E + 05	2.36E + 03	4.70E + 03	1.97E + 02	4.90E + 03	3.34E + 02	3.57E + 03	2.35E + 03
XP_027246859.1	N(4)-(beta-n-acetylglucosaminyl)-l-asparaginase	38261.44	7.12	1.23E + 06	9.43E + 03	1.15E + 04	1.37E + 03	6.19E + 03	1.30E + 03	1.29E + 04	3.62E + 03
XP_027246972.1	Acid ceramidase isoform x2	49761.99	8.82	1.50E + 06	8.16E + 03	1.59E + 04	5.97E + 03	8.56E + 03	6.78E + 03	6.29E + 03	4.22E + 02
XP_027247933.1	Matrix metalloproteinase-19	58055.66	7.71	2.96E + 05	4.19E + 03	1.28E + 04	4.63E + 03	3.88E + 03	5.13E + 02	5.59E + 03	3.10E + 03
XP_027247964.1	Proliferation-associated protein 2g4	43569.39	6.26	3.38E + 05	3.74E + 03	5.78E + 03	6.43E + 02	3.73E + 03	1.88E + 03	3.38E + 03	2.79E + 03
XP_027248157.1	T-complex protein 1 subunit beta	59161.48	6.01	2.12E + 05	5.79E + 03	1.05E + 04	2.92E + 03	4.84E + 03	2.46E + 03	5.70E + 03	3.22E + 03
XP_027248343.1	Endoplasmin	88686.92	4.75	1.27E + 06	1.25E + 04	1.99E + 04	9.46E + 03	5.93E + 03	2.39E + 02	5.95E + 03	3.77E + 03
XP_027248903.1	T-complex protein 1 subunit gamma	60267.3	6.23	3.02E + 05	4.03E + 03	4.27E + 03	1.29E + 03	3.08E + 04	2.08E + 04	3.22E + 03	4.42E + 02
XP_027249017.1	Thrombospondin-3 isoform x1	105716.58	4.4	2.32E + 04	1.44E + 03	3.39E + 03	1.95E + 03	7.44E + 03	1.00E + 03	3.86E + 03	4.02E + 03
XP_027249018.1	Thrombospondin-3 isoform x2	104721.34	4.4	1.79E + 05	7.72E + 03	7.11E + 03	5.20E + 03	8.59E + 03	4.08E + 03	1.81E + 04	1.98E + 04
XP_027249169.1	Protein s100-a6 isoform x1	9841.82	5.3	8.07E + 06	1.67E + 05	1.60E + 04	5.65E + 03	4.65E + 03	3.21E + 03	7.52E + 03	7.43E + 03
XP_027249297.1	Semaphorin-3e	85922.37	7.95	1.44E + 05	2.87E + 03	4.62E + 03	1.20E + 03	1.70E + 03	3.49E + 02	5.89E + 03	4.68E + 03
XP_027249460.1	Extracellular matrix protein 1 isoform x1	62257.78	6.36	1.72E + 04	5.79E + 02	1.28E + 03	1.40E + 02	2.50E + 03	2.60E + 03	6.91E + 02	2.97E + 02
XP_027249462.1	Extracellular matrix protein 1 isoform x3	60267.3	6.98	1.34E + 05	1.89E + 03	1.83E + 04	1.84E + 04	7.95E + 03	4.12E + 03	3.93E + 03	1.67E + 03
XP_027249711.1	Importin-5 isoform x2	118212.37	4.82	8.57E + 04	4.24E + 02	5.88E + 03	9.94E + 02	5.75E + 03	9.18E + 02	6.82E + 03	6.38E + 02
XP_027250030.1	Macrophage migration inhibitory factor	12716.95	6.8	8.91E + 04	4.48E + 03	1.40E + 03	1.11E + 03	6.31E + 03	1.85E + 03	5.03E + 03	3.27E + 03
XP_027251765.1	Glyceraldehyde-3-phosphate dehydrogenase	39367.26	6.67	3.67E + 04	5.91E + 02	2.07E + 03	1.31E + 03	1.19E + 03	2.51E + 02	2.12E + 03	9.94E + 02
XP_027251842.1	Keratin, type ii cytoskeletal 2 epidermal isoform x2	71767.85	6.46	1.50E + 06	9.43E + 03	6.13E + 03	9.39E + 02	3.48E + 03	3.28E + 03	7.69E + 03	3.48E + 03
XP_027252444.1	Keratin, type ii cytoskeletal 1b isoform x2	65243.5	8.51	1.04E + 06	1.63E + 04	1.28E + 03	1.46E + 03	4.72E + 02	5.08E + 02	1.58E + 03	1.56E + 03
XP_027252959.1	Fibronectin isoform x3	264291.46	5.57	3.89E + 04	7.41E + 02	1.21E + 03	3.47E + 02	7.93E + 02	4.26E + 02	2.39E + 04	3.24E + 04
XP_027252960.1	Fibronectin isoform x4	264180.88	5.58	1.52E + 05	1.08E + 04	7.22E + 03	3.35E + 03	2.28E + 04	7.94E + 03	1.34E + 04	7.40E + 03
XP_027252964.1	Fibronectin isoform x8	254228.48	5.77	8.16E + 04	3.45E + 03	6.85E + 03	1.04E + 03	3.82E + 03	1.30E + 03	2.41E + 03	7.61E + 02
XP_027252965.1	Fibronectin isoform x9	251463.92	5.83	2.30E + 05	5.19E + 03	3.07E + 03	7.38E + 02	5.71E + 03	2.44E + 03	4.73E + 03	1.71E + 03
XP_027252966.1	Fibronectin isoform x10	251021.59	5.57	6.16E + 04	2.45E + 03	3.39E + 03	1.18E + 03	3.09E + 03	1.74E + 03	2.11E + 03	2.59E + 02
XP_027252967.1	Fibronectin isoform x11	250911.01	5.59	1.53E + 05	1.89E + 03	4.97E + 03	2.78E + 03	9.17E + 03	2.75E + 03	9.79E + 03	6.14E + 03
XP_027252968.1	Fibronectin isoform x12	240958.61	5.8	1.54E + 05	4.50E + 03	1.46E + 04	1.30E + 04	2.95E + 03	1.30E + 03	6.90E + 03	7.39E + 02
XP_027254100.1	Alpha-enolase isoform x2	47992.67	6	4.97E + 05	1.13E + 04	3.68E + 03	1.84E + 03	1.32E + 04	2.97E + 03	6.68E + 03	2.77E + 03
XP_027255561.1	Basement membrane-specific heparan sulfate proteoglycan core protein isoform x2	485013.53	6.12	7.95E + 05	1.04E + 04	3.00E + 03	2.41E + 02	2.78E + 03	3.88E + 02	2.47E + 03	1.50E + 02
XP_027255877.1	T-complex protein 1 subunit epsilon	59824.97	5.34	3.69E + 05	3.68E + 03	3.49E + 03	2.88E + 03	1.57E + 04	1.21E + 04	9.98E + 03	7.76E + 03
XP_027256703.1	Peptidyl-prolyl cis-trans isomerase c isoform x2	23443.43	7.77	6.34E + 05	1.01E + 04	6.93E + 03	2.65E + 03	2.64E + 03	1.03E + 03	6.95E + 03	4.44E + 03
XP_027259082.1	Transitional endoplasmic reticulum atpase isoform x2	89129.25	5.14	1.47E + 05	5.44E + 03	3.57E + 03	3.16E + 02	1.49E + 03	4.15E + 02	3.68E + 03	1.89E + 03
XP_027259718.1	Myosin-9 isoform x2	216741.11	5.53	1.07E + 05	2.87E + 03	7.70E + 03	3.08E + 03	1.05E + 04	2.62E + 03	1.45E + 04	8.49E + 03
XP_027259782.1	Eukaryotic translation initiation factor 3 subunit l	62368.36	5.93	8.15E + 04	3.27E + 03	1.89E + 03	4.26E + 02	3.65E + 03	3.71E + 03	1.04E + 03	4.07E + 02
XP_027260200.1	Serine protease htra1 isoform x2	53079.46	8.07	3.94E + 05	3.74E + 03	8.62E + 03	2.82E + 03	5.98E + 03	1.59E + 03	1.10E + 04	1.50E + 03
XP_027260201.1	Serine protease htra1 isoform x3	51863.05	8.07	1.01E + 06	1.28E + 04	1.24E + 04	9.94E + 03	7.87E + 03	1.46E + 03	6.79E + 03	3.04E + 03
XP_027260557.1	Lysosome-associated membrane glycoprotein 1	44564.63	6.31	4.37E + 04	5.38E + 03	1.38E + 03	9.38E + 02	7.66E + 02	4.51E + 02	1.87E + 03	2.03E + 03
XP_027260936.1	Inter-alpha-trypsin inhibitor heavy chain h5 isoform x2	105274.25	8.44	2.58E + 05	4.55E + 03	8.44E + 03	3.31E + 03	1.21E + 04	1.76E + 03	1.03E + 04	5.14E + 03
XP_027261047.1	Vimentin	51531.31	5.06	6.48E + 05	8.99E + 03	6.89E + 03	1.49E + 03	6.15E + 03	1.20E + 03	1.08E + 04	1.92E + 03
XP_027263120.1	Phosphoglycerate mutase 1 isoform x2	28087.88	6.67	4.00E + 05	1.14E + 04	3.20E + 03	5.68E + 02	2.80E + 03	1.33E + 03	3.80E + 03	2.32E + 03
XP_027263985.1	Lactadherin isoform x5	54185.28	8.13	7.15E + 04	1.60E + 03	3.87E + 04	2.55E + 04	8.11E + 03	4.41E + 03	1.00E + 04	2.25E + 03
XP_027263987.1	Lactadherin isoform x7	47218.6	8.15	7.42E + 04	3.09E + 03	4.13E + 04	2.40E + 04	2.71E + 04	8.87E + 03	7.45E + 03	2.58E + 03
XP_027264128.1	Elongation factor 1-gamma	48324.42	6.36	4.77E + 05	1.67E + 04	1.37E + 04	6.62E + 03	6.75E + 04	2.97E + 04	1.07E + 04	5.08E + 03
XP_027264138.1	Neutral alpha-glucosidase ab isoform x1	106822.41	5.72	9.50E + 04	3.31E + 03	1.14E + 03	4.22E + 02	8.78E + 02	3.61E + 02	8.97E + 02	3.95E + 02
XP_027264139.1	Neutral alpha-glucosidase ab isoform x2	104389.6	5.64	1.13E + 05	2.05E + 03	2.83E + 03	1.63E + 03	2.97E + 03	5.28E + 02	3.32E + 03	7.89E + 02
XP_027264402.1	Cofilin-1	18356.65	8.22	1.77E + 05	2.49E + 03	1.63E + 04	2.38E + 03	7.33E + 03	3.25E + 03	7.15E + 03	1.52E + 03
XP_027264538.1	Glutathione s-transferase p 2 isoform x2	24549.25	9.15	6.72E + 05	1.45E + 04	5.93E + 03	2.07E + 03	3.97E + 03	7.10E + 02	6.04E + 03	6.45E + 02
XP_027264700.1	Cathepsin d	45117.54	6.54	3.14E + 06	8.52E + 04	4.20E + 03	1.77E + 03	3.69E + 03	2.46E + 02	7.13E + 03	8.45E + 02
XP_027265239.1	Nidogen-1 isoform x3	137785.42	5.07	2.26E + 05	2.16E + 03	8.47E + 03	4.13E + 03	8.31E + 03	1.15E + 03	1.26E + 04	9.76E + 03
XP_027265604.1	Vesicular integral-membrane protein vip36	39367.26	6.46	3.50E + 05	4.55E + 03	4.76E + 03	1.73E + 03	2.83E + 03	2.49E + 02	4.31E + 03	3.65E + 03
XP_027265952.1	Nucleobindin-2 isoform x1	47660.93	4.97	7.63E + 04	2.52E + 03	1.71E + 03	6.58E + 02	4.59E + 03	3.33E + 03	2.87E + 03	6.01E + 02
XP_027265953.1	Nucleobindin-2 isoform x2	46444.52	5.05	1.04E + 06	2.76E + 04	5.90E + 03	4.83E + 02	2.61E + 03	8.63E + 02	9.13E + 03	2.25E + 03
XP_027267107.1	Peptidyl-prolyl cis-trans isomerase b	23885.76	9.59	1.60E + 06	4.71E + 03	3.04E + 03	3.90E + 02	3.38E + 03	2.36E + 02	4.35E + 03	1.07E + 03
XP_027267400.1	Glucosidase 2 subunit beta isoform x2	58055.66	4.41	5.89E + 05	6.68E + 03	5.13E + 03	1.24E + 03	6.16E + 03	2.30E + 03	4.47E + 03	6.21E + 02
XP_027267710.1	Complement c1q tumor necrosis factor-related protein 5 isoform x3	26871.47	5.94	1.02E + 06	3.98E + 04	3.60E + 03	2.52E + 03	3.18E + 03	1.11E + 03	3.28E + 03	9.85E + 02
XP_027267915.1	Neural cell adhesion molecule 1 isoform x4	123852.06	4.71	5.10E + 05	4.32E + 03	8.72E + 03	3.14E + 03	4.99E + 03	2.91E + 03	7.09E + 03	2.69E + 02
XP_027267916.1	Neural cell adhesion molecule 1 isoform x5	93994.87	4.8	5.32E + 04	1.45E + 03	1.36E + 03	5.10E + 02	8.44E + 02	9.77E + 01	2.43E + 03	3.37E + 02
XP_027267917.1	Neural cell adhesion molecule 1 isoform x6	93884.29	4.79	2.57E + 05	8.73E + 03	3.11E + 03	3.85E + 02	1.78E + 03	8.40E + 02	8.69E + 03	2.27E + 03
XP_027267919.1	Neural cell adhesion molecule 1 isoform x8	93773.71	4.8	1.11E + 05	3.30E + 03	5.21E + 03	3.36E + 03	2.83E + 03	3.41E + 03	1.36E + 03	2.69E + 02
XP_027267920.1	Neural cell adhesion molecule 1 isoform x9	80393.26	4.77	2.91E + 05	2.45E + 03	8.57E + 03	3.57E + 03	2.03E + 03	1.79E + 02	5.70E + 03	1.60E + 03
XP_027267921.1	Neural cell adhesion molecule 1 isoform x10	80282.68	4.79	1.15E + 05	2.05E + 03	9.81E + 03	1.40E + 03	3.19E + 03	1.23E + 03	3.04E + 03	3.02E + 02
XP_027268152.1	T-complex protein 1 subunit theta isoform x2	60599.05	5.43	3.22E + 05	4.71E + 02	3.87E + 03	1.01E + 03	1.34E + 04	3.84E + 03	9.09E + 03	5.29E + 03
XP_027268153.1	T-complex protein 1 subunit theta isoform x3	54959.35	5.19	2.89E + 05	4.50E + 03	4.77E + 03	2.10E + 03	1.82E + 03	2.87E + 01	1.98E + 03	8.82E + 02
XP_027269607.1	Beta-glucuronidase isoform x4	72431.34	6.28	1.78E + 05	5.72E + 03	4.30E + 03	3.84E + 03	2.62E + 03	6.07E + 02	1.72E + 03	1.38E + 02
XP_027269616.1	T-complex protein 1 subunit zeta	58719.15	6.46	1.72E + 05	3.09E + 03	6.45E + 03	1.93E + 03	2.54E + 03	1.32E + 03	5.12E + 03	4.02E + 03
XP_027269644.1	Gtp-binding nuclear protein ran isoform x2	23885.76	7.01	5.37E + 05	1.03E + 04	6.36E + 03	2.76E + 03	7.70E + 03	4.69E + 03	6.51E + 03	3.57E + 03
XP_027269956.1	Phospholipase b-like 2	64690.59	5.9	1.10E + 06	4.78E + 04	3.95E + 04	4.71E + 04	1.53E + 04	1.01E + 04	7.36E + 03	1.74E + 03
XP_027270355.1	Transforming protein rhoa	21342.36	5.83	1.56E + 05	1.25E + 03	2.82E + 03	4.60E + 02	2.38E + 03	5.30E + 02	3.50E + 03	1.43E + 03
XP_027270673.1	Pyruvate kinase pkm isoform x1	58719.15	6.69	8.65E + 05	1.64E + 04	1.27E + 04	7.34E + 03	2.17E + 03	6.46E + 02	4.50E + 03	2.64E + 03
XP_027270674.1	Pyruvate kinase pkm isoform x2	58719.15	6.88	1.09E + 06	2.36E + 04	8.46E + 03	3.88E + 03	6.72E + 03	3.48E + 03	5.74E + 03	2.39E + 03
XP_027270675.1	Pyruvate kinase pkm isoform x3	57060.42	8.65	8.09E + 05	2.66E + 04	1.53E + 04	5.98E + 03	4.50E + 03	3.28E + 03	8.36E + 03	2.70E + 03
XP_027270716.1	Lysyl oxidase homolog 1 isoform x2	67012.81	6.57	1.94E + 05	4.71E + 02	7.19E + 03	3.63E + 03	8.20E + 03	2.73E + 03	7.55E + 03	1.64E + 03
XP_027270812.1	Chondroitin sulfate proteoglycan 4	256882.45	5.41	2.63E + 05	5.72E + 03	1.03E + 04	9.31E + 03	1.06E + 04	4.45E + 03	1.45E + 04	6.25E + 03
XP_027271096.1	Beta-galactosidase isoform x3	75859.39	8.58	4.32E + 04	2.36E + 03	1.06E + 03	1.24E + 02	1.81E + 03	7.72E + 02	9.83E + 03	2.59E + 03
XP_027271097.1	Beta-galactosidase isoform x4	73868.91	6.71	3.52E + 05	7.41E + 03	4.27E + 03	5.47E + 02	4.18E + 03	1.49E + 03	5.98E + 03	2.17E + 03
XP_027271619.1	Elongation factor 1-alpha 1	50646.65	7.9	8.78E + 05	4.10E + 04	1.19E + 02	8.16E + 01	5.76E + 01	5.97E + 01	1.89E + 02	1.39E + 02
XP_027271991.1	Malate dehydrogenase, mitochondrial isoform x2	37376.78	8.93	1.35E + 05	4.03E + 03	3.97E + 03	1.35E + 03	3.21E + 03	8.13E + 02	3.77E + 03	8.51E + 02
XP_027272021.1	Plasminogen activator inhibitor 1 isoform x2	44454.04	7.2	4.74E + 06	1.02E + 05	7.43E + 03	2.19E + 03	7.02E + 03	2.94E + 03	9.84E + 03	5.02E + 03
XP_027272044.1	Procollagen c-endopeptidase enhancer 1	51752.47	8.16	2.76E + 05	7.59E + 03	7.26E + 03	3.63E + 03	5.78E + 03	1.27E + 03	5.10E + 03	3.45E + 02
XP_027272591.1	Complement c3	184229.95	6.2	6.09E + 04	2.70E + 03	5.08E + 03	1.33E + 03	1.07E + 04	3.60E + 03	5.28E + 03	1.91E + 03
XP_027272776.1	Laminin subunit beta-1 isoform x5	197499.81	4.78	2.42E + 05	4.71E + 02	6.80E + 03	3.52E + 03	1.80E + 03	5.07E + 02	1.12E + 04	5.03E + 03
XP_027272779.1	Laminin subunit beta-1 isoform x3	186662.75	4.76	1.91E + 05	5.44E + 03	3.96E + 03	4.81E + 02	3.41E + 03	1.03E + 03	6.88E + 03	2.99E + 03
XP_027272780.1	Laminin subunit beta-1 isoform x4	162666.42	4.7	6.79E + 04	1.57E + 03	1.64E + 03	9.69E + 02	2.11E + 03	9.24E + 02	4.12E + 03	5.00E + 02
XP_027273163.1	Laminin subunit gamma-1 isoform x2	177705.6	4.99	1.16E + 05	3.86E + 03	9.84E + 03	9.17E + 03	4.12E + 03	1.37E + 03	4.26E + 03	3.94E + 02
XP_027274751.1	Immunoglobulin superfamily member 8 isoform x3	66902.23	7.94	7.24E + 04	1.28E + 03	2.32E + 03	6.47E + 02	6.75E + 03	4.73E + 03	3.08E + 03	5.51E + 02
XP_027274961.1	Legumain	48435	6.07	3.01E + 06	3.40E + 04	6.31E + 03	1.19E + 03	5.76E + 03	2.85E + 03	6.16E + 03	4.75E + 03
XP_027275654.1	Cathepsin z	33838.15	7.52	3.29E + 06	4.64E + 04	1.44E + 04	6.08E + 03	8.88E + 03	5.11E + 03	5.29E + 03	1.74E + 03
XP_027275687.1	Laminin subunit alpha-5	413024.52	6.49	8.50E + 04	1.33E + 03	8.53E + 03	2.29E + 03	5.54E + 03	3.87E + 03	4.65E + 03	1.58E + 03
XP_027276427.1	Nucleobindin-1 isoform x3	50536.07	5.14	7.26E + 05	8.60E + 03	2.58E + 03	9.22E + 02	5.52E + 03	2.30E + 03	1.06E + 04	6.58E + 03
XP_027277053.1	Protein-glutamine gamma-glutamyltransferase 2	75859.39	5.08	1.08E + 05	3.30E + 03	1.76E + 03	7.39E + 02	1.03E + 03	6.81E + 02	4.04E + 03	2.16E + 03
XP_027278026.1	Thrombospondin-1 isoform x2	129381.17	4.7	1.12E + 06	2.36E + 04	9.32E + 03	3.97E + 03	6.71E + 03	9.85E + 02	7.11E + 03	1.68E + 03
XP_027278163.1	Catalase isoform x1	61373.12	8.29	7.38E + 04	3.74E + 03	2.23E + 03	7.48E + 02	2.00E + 03	1.10E + 03	7.40E + 03	1.58E + 03
XP_027278164.1	Catalase isoform x2	53742.95	7.33	7.48E + 04	1.10E + 03	2.48E + 03	2.46E + 02	2.36E + 03	6.59E + 02	7.45E + 03	8.38E + 02
XP_027279237.1	Matrix metalloproteinase-9	78513.36	5.6	4.31E + 04	2.60E + 03	4.31E + 03	1.59E + 03	1.05E + 04	3.66E + 03	5.71E + 03	2.24E + 03
XP_027279240.1	Lysosomal protective protein	54517.02	5.64	1.34E + 06	0.00E + 00	3.44E + 03	1.21E + 03	6.16E + 03	1.66E + 03	4.86E + 03	3.13E + 03
XP_027279242.1	Phospholipid transfer protein isoform x2	49761.99	5.94	3.95E + 05	2.05E + 03	7.01E + 03	1.74E + 03	2.75E + 03	1.23E + 03	5.27E + 03	1.83E + 03
XP_027279907.1	Insulin-like growth factor-binding protein 4	28087.88	6.78	2.80E + 05	4.71E + 03	2.10E + 03	1.78E + 03	4.60E + 02	1.44E + 02	4.72E + 02	7.07E + 01
XP_027280118.1	Nucleoside diphosphate kinase b	16808.49	7.78	8.71E + 05	8.65E + 03	2.13E + 03	4.30E + 02	5.08E + 03	3.38E + 03	3.93E + 03	7.21E + 02
XP_027280121.1	Nucleoside diphosphate kinase a isoform x1	16808.49	5.96	4.03E + 05	1.02E + 04	1.60E + 04	7.06E + 03	2.90E + 04	1.91E + 04	1.83E + 04	2.18E + 04
XP_027280122.1	Nucleoside diphosphate kinase a isoform x2	12274.62	5	7.94E + 05	4.24E + 03	9.15E + 03	1.12E + 03	5.37E + 03	7.92E + 02	5.05E + 03	1.53E + 03
XP_027280299.1	Emilin-1 isoform x1	112462.1	5.24	1.37E + 05	6.18E + 03	2.13E + 03	6.39E + 02	3.16E + 03	5.94E + 02	9.26E + 03	2.71E + 03
XP_027280300.1	Emilin-1 isoform x2	101625.04	5.09	1.22E + 05	1.25E + 03	5.39E + 03	8.94E + 02	2.52E + 03	6.40E + 02	3.39E + 03	2.41E + 03
XP_027281003.1	Nucleophosmin isoform x2	32290	4.61	9.43E + 05	1.05E + 04	9.38E + 03	7.51E + 03	4.03E + 03	1.60E + 03	5.94E + 03	2.13E + 03
XP_027281136.1	Fatty acid synthase isoform x2	276787.25	5.95	9.64E + 04	2.36E + 03	7.46E + 03	6.20E + 03	7.63E + 03	9.34E + 02	6.81E + 03	3.24E + 03
XP_027281297.1	Lysosomal alpha-glucosidase	104942.51	5.65	6.10E + 05	2.82E + 04	1.64E + 04	9.02E + 03	1.80E + 04	2.88E + 03	4.56E + 03	1.55E + 03
XP_027281318.1	Galectin-3-binding protein	63474.18	5.06	9.51E + 05	2.46E + 04	1.26E + 04	5.04E + 03	8.36E + 03	5.68E + 03	6.46E + 03	3.00E + 03
XP_027281357.1	Septin-9 isoform x4	65464.66	7.16	7.81E + 04	9.63E + 02	1.28E + 04	3.83E + 03	3.51E + 03	2.62E + 03	3.64E + 03	1.17E + 03
XP_027281359.1	Septin-9 isoform x5	37045.04	7.1	7.81E + 04	3.14E + 03	8.26E + 02	3.54E + 02	4.29E + 02	1.56E + 02	6.13E + 02	3.17E + 02
XP_027282087.1	Retinoid-inducible serine carboxypeptidase	50314.9	5.39	4.82E + 05	1.41E + 03	3.12E + 03	1.43E + 03	2.39E + 03	1.85E + 03	2.59E + 03	9.03E + 02
XP_027282154.1	Clathrin heavy chain 1 isoform x3	185225.19	5.48	1.29E + 05	3.56E + 03	3.61E + 04	3.82E + 04	6.45E + 03	2.07E + 03	2.04E + 03	7.87E + 02
XP_027282301.1	C-c motif chemokine 2	15813.25	9.32	3.12E + 06	2.16E + 04	2.03E + 03	4.27E + 02	1.22E + 03	3.98E + 02	4.94E + 03	2.68E + 03
XP_027282549.1	Pigment epithelium-derived factor isoform x2	46223.36	6.43	3.52E + 05	3.74E + 03	7.57E + 03	4.32E + 03	4.18E + 03	1.21E + 03	1.60E + 04	1.49E + 04
XP_027282819.1	Eukaryotic initiation factor 4a-i	44896.37	5.32	1.30E + 05	6.13E + 03	4.71E + 03	1.77E + 03	4.04E + 03	1.25E + 03	5.36E + 03	1.55E + 03
XP_027283255.1	Sparc isoform x4	33838.15	4.75	1.27E + 05	4.71E + 02	2.26E + 03	1.06E + 03	3.57E + 03	8.75E + 02	4.94E + 03	9.27E + 02
XP_027283783.1	Granulins isoform x2	65464.66	5.97	1.15E + 06	2.05E + 04	4.31E + 03	3.70E + 03	3.09E + 03	5.03E + 02	2.42E + 03	3.59E + 02
XP_027284007.1	Keratin, type i cytoskeletal 10 isoform x3	62700.11	5.04	1.14E + 06	3.30E + 04	1.34E + 04	6.51E + 03	1.34E + 04	9.83E + 03	6.90E + 03	7.36E + 02
XP_027284023.1	Complement c1r subcomponent	77960.45	5.71	4.54E + 05	1.17E + 04	1.28E + 04	2.44E + 03	9.64E + 03	2.48E + 03	7.56E + 03	9.74E + 02
XP_027284024.1	Complement c1r subcomponent-like protein	53742.95	6.31	1.27E + 05	4.03E + 03	1.73E + 04	2.46E + 03	1.04E + 04	1.90E + 03	7.97E + 03	2.96E + 03
XP_027284060.1	Triosephosphate isomerase isoform x2	27534.97	6.13	3.21E + 05	4.50E + 03	2.85E + 03	1.18E + 03	7.05E + 03	5.43E + 02	8.88E + 03	1.57E + 03
XP_027284552.1	T-complex protein 1 subunit eta isoform x2	60046.13	7.07	1.99E + 05	9.43E + 02	4.42E + 03	1.16E + 03	5.37E + 03	4.88E + 02	5.42E + 03	1.24E + 03
XP_027286043.1	Transforming protein rhoa isoform x1	21342.36	7.51	3.42E + 04	2.87E + 03	1.12E + 03	1.66E + 02	5.14E + 02	2.04E + 02	3.00E + 03	1.36E + 03
XP_027288083.1	Metalloproteinase inhibitor 1	22448.19	8.84	7.24E + 05	1.45E + 04	9.22E + 03	4.82E + 03	2.12E + 03	4.14E + 02	3.07E + 03	1.06E + 03
XP_027288193.1	V-type proton atpase subunit s1 isoform x2	51199.56	5.52	4.65E + 04	1.42E + 03	1.55E + 03	1.39E + 02	5.38E + 03	2.56E + 03	1.92E + 03	1.26E + 03
XP_027289308.1	Alpha-enolase isoform x2	47992.67	5.85	3.72E + 05	2.87E + 03	5.96E + 03	1.49E + 03	4.81E + 03	1.64E + 03	6.16E + 03	3.51E + 03
